# Incidence of Retinopathy of Prematurity in Neonates with Neonatal Sepsis

**DOI:** 10.34172/aim.2023.38

**Published:** 2023-05-01

**Authors:** Hassan Boskabadi, Majid Abrishami, Nasser Shoeibi, Mahsa Kakavandi, Maryam Moradi, Maryam Zakerihamidi

**Affiliations:** ^1^Department of Pediatrics, Faculty of Medicine, Mashhad University of Medical Sciences, Mashhad, Iran; ^2^Eye Research Center, Mashhad University of Medical Sciences, Mashhad, Iran; ^3^Medical Student, School of Medicine, Mashhad University of Medical Sciences, Mashhad, Iran; ^4^Medical Student, School of Medicine, Iran University of Medical Sciences, Tehran, Iran; ^5^Department of Midwifery, School of Medical Sciences, Tonekabon Branch, Islamic Azad University, Tonekabon, Iran

**Keywords:** Gestational age, Infants, Neonatal sepsis, Preterm, Retinopathy of prematurity

## Abstract

**Background:**

One of the most important complications of premature birth is retinopathy of prematurity (ROP). Sepsis may increase the incidence of this complication. The aim of this study is to compare the incidence of ROP in neonates with and without sepsis.

**Methods:**

In a retrospective case-control study, preterm infants admitted to the neonatal intensive care unit (NICU) of Ghaem hospital from 2014 to 2022 were examined. The case group consisted of 155 preterm infants with definite sepsis (positive blood culture and clinical signs of sepsis) and the control group included 145 preterm infants without sepsis whose maternal and neonatal characteristics were collected; they were examined by a retinologist and evaluated for ROP at 32 weeks or four weeks after birth. Finally, we used the chi-square and the *t* test to compare the two groups.

**Results:**

Out of 155 preterm infants with sepsis, 70% and out of 145 preterm infants without sepsis, 58% had ROP (*P*=0.023). Also, low birth weight, low initial Apgar score and low 5-minute Apgar score were significantly associated with ROP (*P*<0.05).

**Conclusion:**

Based on the results of this study, sepsis is a serious risk factor for ROP. We can reduce its incidence and complication by preventing sepsis in premature infants.

## Introduction

 Due to the advancements in perinatology, the development of assisted reproductive techniques and neonatal care, the probability of premature infant’s survival, especially very low birth weight infants, has increased; However, the rate of morbidity and mortality in them is still high.^1^ Very premature babies have far more problems and need more advanced care. Retinopathy of prematurity (ROP) is one of the most important complications in these babies, which may lead to blindness without follow-up and proper care. In premature infants, ROP develops due to lack of retinal vascularization. It can be a complication due to excessive oxygen therapy.^2^ and causes blindness in about 50 000 infants worldwide each year.^3^ Although many factors have been suggested in the development of ROP, low gestational age, low birth weight and oxygen consumption are definitely involved. In some studies, other possible contributing factors to retinopathy have been also reported such as apnea, mechanical ventilation, anemia, interventricular hemorrhage, sepsis, acidosis, pro-oxidant antioxidant balance, hypovolemia, pneumothorax, bronchopulmonary dysplasia and high arterial level of carbon dioxide.^4-6^

 Another problem with premature infants is the high incidence of neonatal infection, which can lead to neonatal sepsis with high mortality rate; this in turn increases hospitalization time and treatment costs as well as the severity of sepsis complications,^7^ especially in developing countries with limited facilities for infant care.^8,9^ Sepsis is more important in premature and low weight infants because of their immature immune system.^10^ Nosocomial infections are the most common type of infection in the neonatal intensive care unit (NICU),^11^ accounting for between 15% and 20% of cases.^12^ In some studies, neonatal infections have been suggested as a predisposing factor for ROP.^13,14^ Therefore, the aim of this study is to evaluate the rate of ROP in neonates with sepsis and compare it with healthy neonates.

## Materials and Methods

 In this retrospective case-control study, our aim was to compare the incidence rate of ROP in neonates with sepsis and without sepsis, from 2014 to 2022 in Ghaem and Khatam hospitals in Mashhad, Iran.

 Neonates with clinical signs confirming definite sepsis (positive blood culture) were included in the case group and non-infected neonates were chosen as the control group. The case group consisted of 155 preterm infants with definite sepsis (positive blood culture and clinical signs of sepsis) and the control group included 145 preterm infants without sepsis. Clinical signs of sepsis include; lethargy, apnea, respiratory problem, irritability, seizure, need for mechanical ventilation and oxygen therapy, abdominal distension, hypovolemia, meningitis, arthritis, renal failure, cholestatic jaundice and feeding intolerance. Laboratory sings of sepsis include leukocytosis above 25 000, thrombocytopenia (platelet ≤ 150 000), C-reactive protein (CRP) positive ( ≥ 6 mg/dL) and positive blood culture. Definite sepsis must have at least two clinical signs with a positive blood culture. Neonatal information (birth weight, age, sex, gestational age, Apgar score), maternal history (age, pregnancy and delivery problems, type of delivery and parity), infection risk factors and laboratory results were collected and entered in a checklist. Both case and control groups were examined by a retinal specialist at 32 weeks or four weeks after birth.

 All participants in the study underwent retinal examination using a speculum and a lens, which was preceded by a pupil dilator. After the examination and determining the stage and zone, re-examination was performed one to two weeks later, if necessary. There was no need for re-examination in neonates with fully vascularized retina (zone 3).

 Statistical analysis was performed by the SPSS software (IBM SPSS Statistics, version 23). To describe the characteristics of the research units in each of the groups, descriptive statistics including central and dispersion indicators such as mean, standard deviation and frequency distribution were used. Next, the normality of the distribution of quantitative variables was determined by the Kolmogorov-Smirnov test. For inferential statistics, we used parametric tests to compare quantitative variables by case, and if the conditions were not met, we used non-parametric equivalent tests for qualitative variables. We used a univariate logistic regression model to investigate the effect of intervening variables. *P* < 0.05 was considered statistically significant.

## Results

 In this survey, 192 out of 300 neonates (64%) had ROP and 98 neonates (36%) had normal eye examinations. Among neonates with sepsis, the agent was gram-negative in 68% of cases, and gram-positive bacteria in 32% of cases.

**Figure 1:**
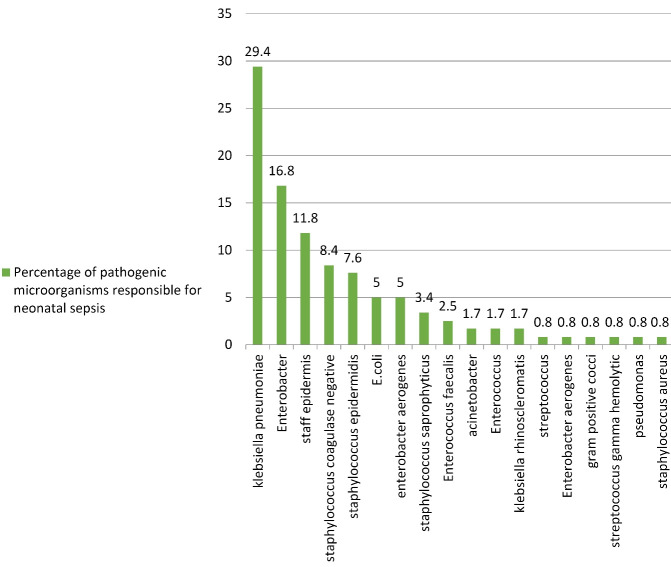
Frequency of Pathogenic Microorganisms Responsible for Neonatal Sepsis

 In the examination of positive cultures, the most frequent pathogenic microorganisms responsible for neonatal sepsis were *Klebsiella pneumonia* (29.4%) and *Enterobacter* (16.8%) (Figure 1).

 The characteristics of infants and their mothers in the two study groups are summarized in Table 1. During this study, generally each infant was examined by an ophthalmologist at least once and a maximum of nine times (mean = 3). According to severity of ROP in the neonates, we found 52 (27.08%) neonates with ROP 0, 77 (40.10%) neonates with ROP 1, 54 (28.12%) neonates with ROP 2, 3 (1.56%) neonates with ROP 3 and 6 (12.3%) neonates with plus disease.

**Table 1:** Comparison of Variables between the Two Groups of Infants with and without Sepsis

**Variables**	**Neonates with sepsis ** **(Mean±SD)**	**Neonates without sepsis ** **(Mean±SD)**	***P *** **Value**^a^
Maternal Age (y)	30.00. ± 4.91	29.22 ± 6.65	0.285
Gestational Age (wk)	30.42 ± 2.49	32.66 ± 2.06	< 0.001
Weight (g)	1288.58 ± 381	1618.91 ± 481	0.025
Apgar 1^st^ minute	5.93 ± 2.26	7.12 ± 1.97	< 0.001
Apgar 5^th^ minute	7.46 ± 1.87	8.60 ± 1.30	< 0.001

SD, Standard deviation. ^a^ *t* test.

 The background characteristics of infants with and without ROP are compared in Table 2.

**Table 2:** Comparison of Variables between the Two Groups with and without Retinopathy of prematurity

**Variables**	**Neonates with ROP (Mean±SD)**	**Neonates without ROP (Mean±SD)**	***P *** **Value**
Maternal age (y)	29.52 ± 6.31	29.61 ± 5.38	0.897
Gravid	2.06 ± 1.10	2.16 ± 1.24	0.532
Gestational Age (wk)	30.87 ± 2.45	32.67 ± 2.31	< 0.001
Birth weight (g)	1385.27 ± 400	1567.95 ± 541	0.003
Apgar score of the 1^st^ minute	6.27 ± 2.28	7.01 ± 1.96	0.004
Apgar score of the 5^th^ minute	7.73 ± 1.76	8.61 ± 1.41	< 0.001

SD, Standard deviation; ROP, Retinopathy of prematurity. ^a^ *t* test.

 In this study, 42.1% of preterm infants without sepsis had normal retinal examination and 57.9% had ROP; 30.3% of preterm infants with sepsis had normal retinal examination and 69.7% had ROP (*P* = 0.023). Using the logistic regression model, the risk of ROP increased with decreasing gestational age, birth weight, and one- and five-minute Apgar scores, and previous sepsis also increased the risk of ROP in the newborns (Table 3). After controlling for birth weight and first-minute Apgar scores in the two groups, the incidence of ROP in the two groups was still significantly different (Table 4).

**Table 3:** Univariate Logistic Regression Analysis

**Variable**	**Univariate Analysis**	
	**OR (95% CI)**	***P *** **Value**
Gestational age	0.744 (0.664–0.834)	< 0.001
Weight	0.999 (0.999–1.000)	< 0.05
First-minute Apgar	0.842 (0.746–0.952)	< 0.01
Fifth-minute Apgar	0.701 (0.589–0.836)	< 0.001
Group	0.617(0.381–0.998)	< 0.05

*P value < 0.05 is considered as significant level.

**Table 4:** Comparison of ROP Incidence between the Two Groups of Neonates with and without Sepsis

**Variables**	**Neonates with Sepsis** **No. (%)**	**Neonates without Sepsis** **No. (%)**	***P *** **Value**
ROP	108 (69.7)	84 (57.9)	0.023
Normal retinal examination	47 (30.3)	61 (42.1)	

^a^ Chi-square. ROP, retinopathy of prematurity.

## Discussion

 Our study shows that the incidence of ROP was 70% in infants with sepsis, and 58% in infants without sepsis (*P* = 0.023). Other studies, such as that of Huang et al have shown that sepsis increases the risk of ROP (*P* < 0.001).^13^ In a meta-analysis, Wang and Tang reviewed 16 studies showing that sepsis increases the incidence of ROP (*P* = 0.011) of any severity; sepsis also increases the risk of severe ROP (*P* < 0.001) (14). Tolsma et al reported a significant association between bacteremia and ROP.^15^ Manzoni et al reported a considerable association between bacterial infection and ROP.^16^ There are various theories as to how sepsis can increase the risk of ROP. For example, microorganisms and their toxins may damage the blood vessel wall. This results in the release of WBCs and their attachment to the blood vessel wall, in return causing micro-thrombosis in the thin wall of retinal blood vessels and blocking them.^14^ Another theory is that inflammatory mediators and growth factors such as interleukin-1β can significantly increase the activity of hypoxia-inducing factor (HIF-1α pathway), which induces and intensifies ROP.^17^

 In our study, the mean gestational age and birth weight were lower in the group with ROP; in several studies, the role of these two factors along with oxygen therapy have been mentioned as the main risk factors for ROP.^11^ Based on several studies, the prevalence of ROP had a significant relationship with birth weight below 1000 g (*P* < 0.001), gestational age under 26 weeks (*P* < 0.001), duration of supplemental oxygen therapy (*P* < 0.001) and 5-minute Apgar score (*P* < 0.005).^18^ Low gestational age and birth weight, and African-American race were predisposing factors for ROP of any severity in another study.^14^ Manzoni also reported that gestational age, birth weight, and duration of supplemental oxygen therapy were associated with ROP.^16^ Finally, despite our efforts in this study, due to the errors and shortcomings of retrospective studies, it is recommended that for achieving better results, a prospective study should be performed.

## Conclusion

 The present study showed that sepsis is a serious risk factor for ROP and by controlling the infection in infants, we may reduce its incidence. Also, we found ROP to be more common in infants who have lower birth weight and lower gestational age.
